# Psychiatric presentations of patients with COVID-19: a retrospective review of 100 consecutive patients seen by liaison psychiatry services

**DOI:** 10.1192/bjo.2021.688

**Published:** 2021-06-18

**Authors:** Yousaf Iqbal, Peter Haddad, Javed Latoo, Majid Alabdulla, Sultan Albrahim, Rajeev Kumar, Ovais Wadoo

**Affiliations:** 1 Hamad medical corporation; 2 Hamad medical corporation, Qatar University

## Abstract

**Aims:**

Coronavirus disease 2019 (COVID-19) is associated with higher rates of psychiatric morbidity due to various factors, including quarantine, social isolation, stigma, financial difficulties and direct and indirect central nervous system impact of severe acute respiratory syndrome coronavirus 2 (SARS-Cov-2).

This study aimed to describe the psychiatric morbidity of patients with COVID-19 referred to liaison psychiatry services in Qatar.

**Method:**

This study was a retrospective review of patient records of the first 100 consecutive SARS-Cov-2 positive patients referred to liaison psychiatry services. The study was approved by the Hamad Medical Corporation Institutional Review Board (IRB) (MRC-05–072). Data were analysed using descriptive statistics.

**Result:**

The majority (n = 92) of 100 included patients were male and median age was 43 years. Patients were of diverse background with majority of South Asian (Indian, Pakistani, Bengali, Nepalese, and Afghan) (n = 60), followed by Qatari (n = 18) background. Mean length of hospital stay was 26.51 days.

35 patients had severe or critical COVID-19 pneumonia, and 67 had at least one underlying physical comorbidity. Significant psychosocial stressors other than positive SARS-Cov-2 status, including lockdown, quarantine, finances and relationships issues were identified in 48 patients.

A total of 35 patients had a positive past psychiatric history, out of which 17 were on maintenance psychotropic medications. Insomnia was the commonest psychiatric symptom (n = 65), followed by anxiety (n = 52), agitation (n = 42), depression (n = 39), changes in appetite (n = 32) and irritability (n = 30). The principal psychiatric diagnoses made were delirium (n = 29), acute stress reaction or adjustment disorder (n = 25), depression (n = 16), mania (n = 15), anxiety (n = 14), non-affective psychosis (n = 13), and dementia (n = 6). Approximately half of the patients with mania or non-affective psychosis had it as their first-onset disorder.

**Conclusion:**

SARS-CoV-2, in both symptomatic and asymptomatic patients, is associated with a wide range of psychiatric morbidity which emphasizes clinicians’ vigilance for psychiatric symptoms. Insomnia was the commonest neuropsychiatric symptom which may have clinical practice and potential preventive strategies implications.

Delirium, the commonest diagnosis in the study carries high morbidity and mortality and may reflect SARS-Cov-2 propensity to affect the brain directly and indirectly through a cytokine storm, organ failure, and prothrombotic state. Patients can also present with new-onset mania or non-affective psychosis. It is noteworthy that about two-thirds of the patients had no past psychiatric history.

This study, along with expanding body of evidence may assist with resource allocation and liaison psychiatry services planning. It also underscores the importance of designing future studies to better understand longer-term psychiatric sequelae of COVID-19.

